# We Need to Talk About Quality of Life with Cancer Patients: Primum Non Nocere in Oncology

**DOI:** 10.3390/medicina61050918

**Published:** 2025-05-19

**Authors:** Vlad Bogin

**Affiliations:** Suite 300, Preston Commons Center, 8117 Preston Road, Dallas, TX 75225, USA; vbogin@cromospharma.com

**Keywords:** quality of life, oncology, cancer treatment, primum non nocere, functional status, drug toxicity, number needed to harm, shared decision making, palliative care, patient-centered care, end-of-life care

## Abstract

The Hippocratic principle primum non nocere, or “first, do no harm”, serves as a vital lens through which to re-evaluate modern oncology practices. While recent advances such as immunotherapy, targeted agents, and precision medicine have transformed cancer care, these treatments are not without risk. Even with improved tolerability, they may still lead to substantial toxicities, particularly in frail patients with advanced cancer. The pursuit of survival often overshadows the patient’s quality of life, with aggressive interventions frequently continuing beyond the point of meaningful benefit. This perspective article argues for a more individualized and ethically grounded approach to cancer treatment, emphasizing the careful assessment of each patient’s clinical status, values, and goals. By integrating geriatric and palliative assessments, improving shared decision making, and moving away from a default treatment-at-all-costs mindset, clinicians can better align care with what truly matters to patients. Honoring primum non nocere in oncology means not only extending life when appropriate but ensuring that life remains worth living.

## 1. Introduction

The foundational principle primum non nocere, or “first, do no harm”, remains deeply relevant to oncology today, particularly in the context of treating patients with advanced or incurable cancer. As the field continues to evolve with breakthroughs in immunotherapy, targeted agents, and molecular diagnostics, there is a growing need to reassess how these innovations align with patient values and lived experiences. Although many of these therapies are associated with improved tolerability compared to traditional cytotoxic chemotherapy, they are not benign. Immune-related adverse events, cumulative fatigue, organ-specific toxicities, and complex monitoring requirements can still impose significant burdens, especially in older or functionally compromised individuals.

Cancer care has traditionally emphasized survival endpoints such as overall survival (OS) and progression-free survival (PFS) as the gold standards of therapeutic success. However, this focus on numerical outcomes can sometimes obscure the impact of treatment on patients’ quality of life. Prolonging life may come at the cost of functional decline, emotional distress, and financial hardship. Despite increased attention to patient-reported outcomes (PROs), clinical decision making often defaults to aggressive treatment, even when the expected benefit is marginal. This is particularly true in advanced-stage disease, where continuing therapy beyond a clinically meaningful point may result in greater harm than benefit.

In this context, careful patient selection becomes critical. Assessing not only disease characteristics but also functional status, comorbidities, geriatric vulnerabilities, social support, and individual goals can help clinicians determine whether a proposed treatment is likely to result in a net benefit. Comprehensive geriatric assessments and structured decision-making tools should be routinely employed to identify patients who may be harmed more than helped by aggressive interventions. This approach supports the ethical obligation to tailor care according to the patient’s unique clinical profile rather than reflexively following disease-based algorithms.

Furthermore, the prevailing ethos within oncology often equates action with hope. The instinct to continue treatment, even when its utility is questionable, can delay necessary conversations about prognosis, goals of care, and alternative pathways such as supportive or palliative interventions. In many cases, stepping back from disease-directed treatment is not a failure, but a strategic and compassionate decision aligned with the patient’s priorities.

This article argues for a recalibration of oncologic decision making, one that places greater weight on the patient’s definition of a meaningful life rather than survival alone. By integrating ethical reflection, recent data, and practical frameworks for communication and care planning, we propose a more person-centered model for modern oncology. In honoring the principle “do no harm”, we must ensure that treatment does not erode the experience of living with cancer.

## 2. Functional Status vs. Disease Stage: Rethinking Prognostic Indicators

In oncology, cancer staging has traditionally served as the primary determinant of therapeutic strategies, offering a standardized method for assessing disease progression and informing treatment selection. While staging remains essential for clinical decision making, growing evidence suggests that functional status—defined as a patient’s capacity to perform activities of daily living—may be a more powerful and clinically relevant prognostic marker, particularly in older adults or those with advanced disease.

Patients with high functional status, such as those categorized as having an Eastern Cooperative Oncology Group (ECOG) Performance Status of 0 or 1, generally tolerate systemic therapy more effectively and experience fewer severe toxicities compared to those with diminished functional reserve [1]. In contrast, initiating aggressive cancer therapies in patients with poor performance status is frequently associated with accelerated functional decline, loss of independence, and deterioration in psychosocial well-being, often without offering a meaningful survival benefit.

Objective assessment tools have been developed to guide this aspect of care. Instruments such as the ECOG Performance Status, the Karnofsky Performance Scale, the Geriatric 8 (G8) screening tool, and the Comprehensive Geriatric Assessment (CGA) provide structured frameworks to evaluate functional domains including mobility, nutrition, cognition, and social support. Incorporating these metrics into routine oncologic evaluation can enhance risk stratification, optimize treatment selection, and reduce the likelihood of overtreatment in vulnerable populations.

Studies demonstrating that functional impairment independently predicts increased morbidity, higher chemotherapy toxicity, and reduced overall survival [2] further support this need for structured assessment. A chemotherapy-related decline in physical function among older adults has been shown to exceed the functional deterioration expected from cancer progression alone, raising critical concerns about the appropriateness of aggressive treatment in this population [3].

Moreover, functional impairment is not only a physical concern but also a psychological one. It is strongly associated with elevated rates of depression in cancer patients, with performance status, fatigue, and symptom burden identified as key predictive factors [4]. This underscores the importance of a holistic, function-centered evaluation that incorporates mental health and psychosocial well-being.

Finally, emerging data on patient preferences reinforce the clinical relevance of functional status in treatment planning. In studies involving individuals with early-stage lung cancer, patients consistently ranked the preservation of independence and daily functioning above prolongation of life or concerns about recurrence [5]. Many were willing to accept higher procedural risks if it meant maintaining autonomy, suggesting that treatment paradigms should move beyond tumor-centric metrics and instead prioritize outcomes that reflect what patients value most.

## 3. Drug Toxicities: The Fine Line Between Treatment and Harm

Cancer therapeutics, while increasingly precise, continue to rank among the most toxic regimens in contemporary medicine. The therapeutic efficacy of chemotherapy, targeted therapies, and immunotherapy must be carefully weighed against their substantial potential for adverse effects, which can range from transient and manageable to permanent and life altering. Although advances in drug development have improved tolerability for many patients, significant toxicities persist and may, in some cases, outweigh the clinical benefit, particularly in vulnerable populations such as older adults or patients with limited functional reserve. These risks highlight the need for systematic toxicity monitoring, individualized treatment planning, and early implementation of mitigation strategies.

Cardiotoxicity remains one of the most extensively studied and clinically significant adverse effects in oncology, particularly with anthracyclines such as doxorubicin. These agents, while effective, induce myocardial damage through mechanisms including free radical formation and DNA intercalation, resulting in a well-characterized risk of dose-dependent cardiomyopathy. Clinical studies suggest that symptomatic heart failure occurs in approximately 5–10% of patients receiving anthracycline-based therapy and suggest that subclinical left ventricular dysfunction is likely under-recognized [6]. Targeted agents such as bevacizumab and VEGF tyrosine kinase inhibitors are associated with increased cardiovascular risks, including arterial thromboembolic events, hypertension, and congestive heart failure, warranting careful cardiovascular assessment and ongoing monitoring during treatment [7].

Neurotoxicity presents another critical challenge. Platinum-based compounds, particularly cisplatin and oxaliplatin, induce cumulative, dose-dependent peripheral neuropathy characterized by distal sensory deficits, paresthesia, and impaired proprioception [8]. These symptoms may persist long after cessation of therapy and are frequently irreversible, significantly impairing daily function and quality of life. Taxanes such as paclitaxel and docetaxel also cause debilitating neuropathies, including dysesthesia and motor weakness, which may confound long-term survival [9]. Immune checkpoint inhibitors have further expanded the spectrum of neurotoxicity, with rare but serious immune-mediated syndromes including Guillain–Barré syndrome, myasthenia gravis, and autoimmune encephalitis increasingly reported [10].

Renal and otologic toxicities are particularly relevant for cisplatin-based chemotherapy. Cisplatin accumulates in renal tubular epithelial cells, resulting in acute tubular necrosis and a high incidence of acute kidney injury, observed in up to 20–30% of treated patients [11]. Nephrotoxicity is compounded by electrolyte disturbances and, in some cases, progression to chronic kidney disease. Concurrently, cisplatin-induced ototoxicity, which primarily affects high-frequency hearing, results from apoptosis of cochlear hair cells and is often irreversible. This is especially concerning in pediatric and adolescent populations, where such damage may impair neurocognitive development and social functioning [12].

Hematologic toxicities remain a defining feature of cytotoxic therapies. Myelosuppression, manifesting as neutropenia, thrombocytopenia, and anemia, can lead to treatment delays, infections, bleeding, and fatigue. Neutropenia, a frequent complication of many chemotherapeutic agents, necessitates prophylactic or reactive use of granulocyte colony-stimulating factors to mitigate the risk of life-threatening infections [13]. Thrombocytopenia, common with alkylating agents and taxanes, increases bleeding risk and often requires dose modifications. Anemia, whether from bone marrow suppression or inflammation, contributes to reduced physical performance and worsened fatigue. Notably, immune checkpoint inhibitors have been linked to autoimmune hematologic complications such as hemolytic anemia, aplastic anemia, and immune thrombocytopenia, conditions that may require corticosteroids or other immunosuppressive agents for resolution [14].

Gastrointestinal and hepatic toxicities also pose substantial barriers to treatment tolerability and continuity. Irinotecan-induced diarrhea, a result of both acute cholinergic effects and delayed mucosal injury, often necessitates dose reductions or discontinuation [15]. Fluoropyrimidines such as 5-fluorouracil and capecitabine are commonly associated with mucositis and palmar–plantar erythrodysesthesia (hand–foot syndrome), leading to pain, desquamation, and functional impairment. Moreover, immune-related colitis and hepatitis induced by checkpoint inhibitors may evolve rapidly and require immunosuppressive interventions, including corticosteroids, infliximab, or mycophenolate mofetil, to prevent permanent organ damage [16].

Given the breadth and severity of these toxicities, oncology providers must ensure that patients are fully informed about potential risks before initiating treatment. Structured toxicity risk assessment tools and comprehensive pretreatment evaluation should be standard components of oncology practice, particularly when treating patients with limited functional reserve or shortened life expectancy. Toxicities not only affect physical health but may also lead to emotional distress, prolonged hospitalizations, and impaired long-term functioning. For some patients, especially those with advanced disease, the burden of toxicity may outweigh potential therapeutic gain.

Shared decision making should, therefore, include explicit discussions of anticipated toxicities, their likelihood, and their potential reversibility. Evidence-based counseling, rooted in both clinical data and patient values, allows individuals to make informed choices regarding what level of toxicity they are willing to accept. For patients with limited prognosis, these conversations become even more critical, enabling a transition from disease-modifying therapy to comfort-focused care when appropriate.

## 4. The Problem of Number Needed to Harm (NNH) in Oncology

In oncology, clinical decision making frequently involves a complex evaluation of therapeutic benefits relative to treatment-related risks. Epidemiologic metrics such as the number needed to treat (NNT), defined as the number of patients who must receive therapy for one to benefit, and the number needed to harm (NNH), representing the number of patients treated for one to experience a serious adverse event, are commonly used in many areas of medicine to guide these decisions. However, in oncology, where the magnitude of survival gains is often modest and treatment toxicities are frequent and significant, the interpretation and application of these metrics are far less straightforward.

In cardiology, for example, statins have an NNT of approximately 50 to 100 to prevent one major cardiovascular event over five years, while the NNH for severe adverse effects such as rhabdomyolysis is between 200 and 300, making the benefit-to-risk ratio favorable [17]. Similarly, in the management of acute exacerbations of chronic obstructive pulmonary disease, antibiotics have a number needed to treat (NNT) of 12 to prevent clinical deterioration, whereas the number needed to harm (NNH) for a serious allergic reaction is approximately 1 in 2000, supporting a favorable benefit–risk profile and facilitating confident therapeutic decision making [18].

By contrast, oncology frequently operates in a therapeutic space characterized by high NNTs and relatively low NNHs. Many treatments for advanced malignancies result in only marginal improvements in median survival, often measured in weeks or months, while exposing many patients to considerable toxicity. In the context of advanced pancreatic cancer, gemcitabine has been shown to increase survival by approximately eight weeks over best supportive care, yet nearly one-third of patients experience severe grade 3 or 4 toxicities [19]. This narrow margin between benefit and harm challenges the traditional clinical rationale for initiating treatment and requires more nuanced decision making.

The difficulty in applying these metrics to oncology stems in part from the reliance on surrogate endpoints such as progression-free survival or radiographic response rates, which do not always correlate with improved overall survival or enhanced quality of life. Unlike fields where treatment goals are often curative or preventive, oncology must contend with the reality that many therapies offer only transient disease control without addressing the underlying burden of symptoms or preserving function. Additionally, clinical trials may under-report or inadequately assess patient-reported outcomes, which are essential to understanding how patients experience both benefit and harm [20].

Regulatory frameworks may also contribute to this imbalance. In other medical specialties, new therapies are typically approved only after demonstrating both meaningful clinical efficacy and acceptable safety. In oncology, however, many agents receive accelerated approval based on surrogate endpoints, with limited data on long term toxicity and real-world effectiveness. This can lead to widespread clinical use of therapies before their full risk profile is understood, leaving clinicians and patients to navigate substantial uncertainty [21].

Given these complexities, the NNT and NNH should be used in oncology not as rigid decision thresholds but as tools to guide informed, values-based discussions. Clinicians must help patients weigh trade-offs in the context of their goals, functional status, and risk tolerance. For some, modest survival gains may be meaningful; for others, preserving their quality of life may take priority. In all cases, transparent communication and alignment with patient values should drive treatment choices.

## 5. The Overuse of Chemotherapy at the End of Life

Emerging evidence has drawn attention to the persistent overutilization of systemic chemotherapy in patients with advanced, incurable malignancies during the final stages of life. A recent large-scale analysis of health records from 78,446 adults with metastatic breast, colorectal, non-small-cell lung, pancreatic, kidney, and urothelial cancers revealed that the administration of systemic therapy near the end of life conferred no significant survival advantage [22]. These findings reinforce growing concerns that aggressive treatment in this setting may serve primarily to prolong the dying process, often at the expense of patient comfort and dignity.

Multiple factors contribute to this pattern of overtreatment. Clinicians may struggle to transition patients from disease-directed therapy to palliative care, due, in part, to therapeutic optimism, emotional attachment, or discomfort with prognostic disclosure. Simultaneously, patients and their families may equate the discontinuation of active treatment with abandonment or a loss of hope, despite evidence that continued chemotherapy may reduce, rather than improve, the quality of life in the terminal phase of illness [23]. Systemic drivers, including reimbursement models and institutional incentives, may further perpetuate the inclination to offer treatment, even when clinical benefit is unlikely.

From a healthcare utilization perspective, the administration of chemotherapy near the end of life is associated with increased resource consumption and decreased quality-adjusted survival. Patients who receive systemic therapy in their final weeks are more likely to be hospitalized, undergo invasive procedures, and experience financial hardship instead of spending time in familiar environments surrounded by loved ones [24]. In contrast, early integration of palliative care has demonstrated improvements in both quality and duration of life, facilitating goal-concordant care, reducing unnecessary interventions, and enabling informed decision making [25].

Professional guidelines reflect the urgency of addressing this issue. The American Society of Clinical Oncology and the European Society for Medical Oncology have issued recommendations discouraging the use of chemotherapy in patients with terminal cancer who are unlikely to derive meaningful benefits [26]. Despite these efforts, real-world data consistently show that such guidelines are not uniformly implemented, with many patients continuing to receive treatment within days of their death.

Addressing chemotherapy overuse near the end of life requires both systemic change and a cultural shift in oncology. Clinicians must be equipped to hold timely, honest conversations about prognosis and care goals. Using structured communication tools and validated assessments can help identify patients unlikely to benefit from further treatment. Ultimately, care should align with patient values, ensuring that end-of-life decisions reflect compassion, clarity, and respect for individual preferences.

## 6. Outliers and the Mirage of the “Miracle Response”

The continuation of aggressive cancer treatment is frequently rationalized by reference to rare individuals who achieve exceptional therapeutic responses. These so-called outliers, while scientifically noteworthy, constitute statistical anomalies and should not inform routine clinical decision making for the broader oncology population. The appeal of the “miracle response” may foster unrealistic expectations, leading clinicians and patients to pursue therapies that offer limited survival benefits for the majority, while imposing significant toxicity and compromising quality of life.

Pancreatic adenocarcinoma illustrates this dilemma well. With a five-year survival rate of approximately 10 percent, it remains one of the most lethal solid tumors [27]. Nevertheless, many patients with advanced pancreatic cancer continue to receive systemic therapy during the terminal stages of illness in the hope of becoming one of the rare individuals who defy the odds. Real-world evidence, however, consistently indicates that such outcomes are uncommon. Treatment in the final weeks of life is more often associated with adverse events, increased hospitalization rates, and reduced quality of life rather than clinically meaningful life extension.

The persistence of this treatment pattern is influenced by multiple factors, including cognitive and emotional biases among providers. Physicians may feel compelled to continue offering active treatment due to a reluctance to acknowledge therapeutic limitations, fear of appearing to abandon the patient, or discomfort initiating end-of-life discussions. Simultaneously, patients and families may perceive the cessation of therapy as a form of surrender, prompting continued pursuit of further treatment lines even when the probability of benefit is exceedingly low [28].

Although the biological insights gleaned from exceptional responders are of legitimate scientific interest and may inform future therapeutic development, such cases should not define current standards of care. Clinical guidelines and treatment decisions must be grounded in robust data that reflect the likely outcomes for the typical patient. Anchoring clinical reasoning to rare outliers can distort risk perception and promote interventions that ultimately detract from patient well-being.

## 7. Moral and Philosophical Dilemmas: The Fear of Taking Away Hope

One of the most ethically and emotionally complex challenges in oncology involves determining when to transition the focus of care from disease-modifying treatment to palliation. Clinicians frequently hesitate to initiate conversations about prognosis and the limits of therapy, concerned that doing so may extinguish a patient’s sense of hope or be perceived as abandonment. Research suggests that this reluctance is not merely an individual failing but reflects a shared cultural aversion to suffering and death, affecting both patients and healthcare providers. Patients may describe their cancer journey as a battle, where discontinuing treatment is seen as surrender, while physicians may struggle to frame the decision as a deliberate and compassionate course correction rather than a concession of defeat. Such framing can result in overtreatment, increased toxicity, and a measurable decline in patient quality of life [29].

Prognostic uncertainty further complicates this process. Many clinicians fear being wrong about life expectancy or worry that disclosing a poor prognosis will deprive patients of optimism. However, the concept of hope in the context of serious illness is frequently misunderstood. While hope is initially tied to the possibility of a cure or long-term remission, its meaning often evolves as patients approach the end of life. Studies have shown that individuals facing advanced illness frequently shift their hopes toward comfort, the preservation of dignity, and time with loved ones [30]. Providers who are attuned to this shift can play a pivotal role in helping patients reframe their goals and make decisions that are aligned with their values and lived priorities.

## 8. A Shift Towards Honest Conversations and Palliative Care

Conversations regarding prognosis, therapeutic limitations, and transitions to supportive or palliative care remain among the most challenging responsibilities in oncology. Yet when such discussions are avoided or postponed, patients and their families may be left unprepared for the progression of illness. This lack of preparedness can lead to the continuation of burdensome medical interventions that are not aligned with the patient’s goals or preferences, ultimately diminishing the quality of the final phase of life.

Facilitating these conversations requires structured, deliberate communication strategies that emphasize clarity, empathy, and shared decision making. The SPIKES protocol is a widely endorsed framework for delivering serious news in oncology. It emphasizes the importance of setting up a private and respectful environment, assessing the patient’s understanding of their condition, determining the extent of information they wish to receive, and delivering medical information compassionately and honestly [31]. A core tenet of this approach is to validate emotional responses while guiding patients toward a care plan that reflects their values and priorities.

In addition to SPIKES, the Ask Tell Ask method offers a concise model that promotes patient engagement and iterative understanding. This technique begins by asking patients what they already know and what they are most concerned about, followed by providing medical information that is responsive to their level of readiness. The conversation is then redirected back to the patient to confirm their understanding and to clarify their goals moving forward. These frameworks represent a departure from traditional disease-centered models of care, instead emphasizing individualized outcomes, symptom control, and alignment with patient-defined objectives [32].

Despite the availability of these communication tools, hesitancy to initiate conversations about prognosis and palliative transitions remains pervasive among healthcare professionals. This hesitancy underscores the need for systematic training in communication skills as a core component of medical education. Competency in discussing treatment goals, functional decline, symptom burden, and end-of-life care should be cultivated alongside clinical knowledge to ensure that patients are guided through their illness with honesty, compassion, and respect.

## 9. Conclusions

The care of patients with advanced cancer presents profound clinical and ethical challenges. While oncology has achieved remarkable progress in extending life through innovative therapies, these advances can come with significant burdens, particularly for individuals with limited functional reserve or terminal illness. The imperative to preserve life often stems from a deep and compassionate commitment to patients, yet it may sometimes unintentionally lead to interventions that prolong suffering rather than meaningfully improve outcomes.

The tendency to continue aggressive treatment late into the disease trajectory is rarely a result of negligence or disregard for patient well-being. Rather, it reflects the emotional complexity of oncology, where hope, uncertainty, and the desire to offer every possible chance can shape decision making. Physicians, patients, and families alike grapple with the tension between doing something and acknowledging the limits of medicine. In this context, reframing therapeutic success to include not only the duration of life but also its quality, comfort, and alignment with patient values becomes essential.

Early and transparent conversations about prognosis, functional status, and treatment limitations allow for more informed and personalized choices. Structured communication approaches, such as the SPIKES protocol and the Ask Tell Ask method, can support clinicians in navigating these discussions with clarity and empathy. Training in these skills should be integrated into oncologic education, ensuring that clinicians feel equipped to guide patients through emotionally difficult decisions.

System-level reforms must also support this shift in perspective. Policies and reimbursement structures that prioritize quality-of-life outcomes, rather than defaulting to high-intensity interventions, can help clinicians offer care that is both evidence-informed and ethically sound. Regulatory evaluations of new therapies should include not only survival endpoints but also real-world effectiveness, functional impact, and patient-reported outcomes.

Ultimately, a more nuanced and humanistic vision of success in oncology is needed, one that honors not only how long patients live but how well they live. Shared decision making, respect for individual goals, and thoughtful integration of palliative care are not signs of giving up but of recognizing the full scope of our obligation to do no harm. These values form the foundation of a model of care that respects both the science and the humanity of cancer medicine.

As this article represents a perspective, it reflects a personal interpretation of current clinical and ethical challenges, which may vary depending on the practice setting, cultural context, and patient population.

## Data Availability

No new data were created or analyzed in this study.
